# Stochastic gene expression and chromosome interactions in protecting the human active X from silencing by *XIST*

**DOI:** 10.1080/19491034.2020.1850981

**Published:** 2021-01-18

**Authors:** Barbara R. Migeon

**Affiliations:** Departments of Genetic Medicine and Pediatrics, The Johns Hopkins University, Baltimore, MD, USA

**Keywords:** X chromosome inactivation, protecting the active X from XIST silencing, stochastic gene expression, 3-D nuclear interactions, X/autosome interactions, 19p trisomies

## Abstract

Mammals use X chromosome inactivation to compensate for the sex difference in numbers of X chromosomes. A relatively unexplored question is how the active X is protected from inactivation by its own XIST gene, the long non-coding RNA, which initiates silence of the inactive X.  Previous studies of autosomal duplications show that human chromosome 19 plays a critical role in protecting the active X. I proposed that it genetically interacts with the X chromosome to repress XIST function on the future active X.  Here, I show that the type of  chromosome 19 duplication influences the outcome of the interaction: the presence of three chromosome 19s is tolerated whereas duplications affecting only one chromosome 19 are not. The different outcomes have mechanistic implications for how chromosome 19 interacts with the future active X, pointing to a role for stochastic gene expression and possibly physical interaction.

## Introduction

X inactivation is the mechanism used by eutherian mammals to compensate for the sex difference in their number of X chromosomes [1]. The common feature in human diploid cells is silencing of all X chromosomes, except one. This is accomplished by the long non-coding RNA, *XIST* [2]. *XIST* is a potent chromosome inactivator, capable of silencing any chromosome from which it is expressed [3]. The details of X inactivation vary among mammals: abundant evidence indicates that this developmental program in humans and many other mammals differs from that occurring in rodents [4–7]. Species also differ as to how the active X in both males and females is protected from silencing by *XIST* [6]. Unlike rodent genomes, humans and most other mammalian genomes do not include a functional antisense to *XIST* [8,9]. While much effort in the field has focused on how the inactive X chromosome is inactivated, an underexplored question is how the human active X chromosome is protected from inactivation by its own *XIST* locus? The mechanisms for this process are not well defined in any species [5,10]

It seems likely that mice use Tsix, the non-coding RNA that is antisense to *Xist*, to protect their future active X from silencing by Xist RNA [10,11], but how *Tsix* exerts this function remain obscure [11–13]. The *Xist* gene on the active X of mice is repressed in maternal oocytes before conception; maternal *Xist* repression may be initiated by *Tsix*, in conjunction with the heterochromatin protein complex *PRC2*, which plays an important role in the process [13]. Regardless, the maternal X remains active when *Xist* transcription is upregulated in the trophectoderm, turning off the paternal X [11,13]. Unlike rodents, and like most eutherian mammals, human *XIST* is not imprinted during oogenesis to remain active, and the human truncated *TSIX* locus plays no role in X–inactivation [9,14].

Two observations point to a role for dosage sensitive gene(s) on the short arm of human chromosome 19 to assure that one X remains active in both males and females. First, the extra set of autosomes in human triploid embryos (69,XXX or 69,XXY) protects two X chromosomes from silencing unlike the single X, protected in diploid cells [15]. Second, all 20 clinically recognizable human autosomal trisomies (47, XX), have a single active X [15,16] which means that none of these triplicated autosomes played a role in protecting the second X from inactivation. This implicates trisomy 19 or 1, the only autosomal trisomies that were not studied because they all die before implantation. Examining partial trisomies of 1 and 19 among human liveborn using the DECIPHER database [17] – which maps genetic variants in tens of thousands of patients and correlates them with phenotype – we identified the short arm of chromosome 19 (19p) as the relevant autosome, based on sex differences in duplications [6].

Duplicating the putative *XIST* repressor should not be lethal in fetal males; as they have only one X chromosome, it is the only one that can be silenced, and so males can tolerate extra repressor. However, duplication of this region in females could provide enough repressor to keep both X’s active, which is known to be lethal [18]. Chromosome 19 was the only chromosome with the expected sex difference in duplications [6]. That only males had a duplication, indicates that females with similar variants must not have survived *in utero*. Of interest, the USC browser shows that this ~ 8 MB region of chromosome 19p is surprisingly well conserved on one chromosome in all eutherian mammals, except rodents, where the region is split among four different chromosomes [7].

Here, I have analyzed new cases of 19p duplications in the DECIPHER database [17]. My analysis indicates that not all of them are alike as only interstitial duplications that affect one chromosome 19p are associated with preimplantation loss of females; duplications of 19p caused by unbalanced translocations are not. The difference between the two types of duplications points to stochastic gene expression as a crucial event in X chromosome protection.

## Results & discussion

Since publication of our original search in 2017 [6], five females with 19p duplications have been added to the DECIPHER database [17] – four with sufficient data to interpret [19–22]. The four females were studied in the 1990s by chromosome banding or FISH analysis, before accurate genome mapping became available; therefore, they are not suitable for breakpoint analysis and duplication mapping. On the other hand, three of the four females differ from the usual *in situ* duplications, as they are due to unbalanced translocations between two different autosomes (Table 1). Because the three females with 19p duplications (*394,820, 395,096 and 395,557* in the DECIPHER database) have unbalanced translocations that were balanced in a parent, they had three copies of some 19p genes, each on a separate chromosome (Tables 1 and Tables 2) The presence of this critical region of chromosome 19 on three separate chromosomes, rather than duplicating the region on one chromosome, enables some of these females to survive gestation. Comparing the two kinds of 19p duplications provides insights into the mechanisms for the repression of the *XIST* locus on the future active X.

**Table 1.** Three females with duplications due to unbalanced translocations involving chromosome 19p

Decipher patient #	Sex	Kind of duplication	Size of 19p duplication	Inheritance	Phenotype
394,820	F	Unbalanced translocation *3;19*	13.88 MB^a^	Paternal balanced	Facial dysmorpia, microcephaly, deafness, short, renal hypoplasia, intellectual disability. *Living at > 30 yrs*
395,096	F	Unbalanced translocation *4;19*	6.89 MB^a^	Maternal balanced	Facial dysmorphia, microcephaly, atrial septal defect, intellectual disability, *Died at age 3 yrs*
395,557	F	Unbalanced translocation *4;19*	23.65 MB^a^	Paternal balanced	Facial dysmorphia, microcephaly, adrenal hypoplasia, renal hypoplasia, ectopic anus. small for age (“*Wolf-Hirschorn Syndrome”)* *Died 33 wks gestation*

^a^Size uncertain because determined by chromosome banding^.^

**Table 2.** Stochastic interactions between X-linked XIST and its repressor(s) on chromosome 19p, with respect to the number of 19p chromosomes in the interaction and, excessive repressor delivered

	Number of Interacting Chromosome 19p		
Female Karyotype	One 19p	Two 19p	Three 19p
46 XX *Two normal 19ps*	XX XX XX XX	XX XX XX XX	NA
46XX (19p dup interstitial) *One normal 19p* *One duplicated 19p*	XX XX XX XX *50% excess*	XX XX XX XX *100% excess*	NA
46XX, (−3,+der(3)t(3;19) (q29;p13.2)pat *Two normal 19ps* *One 3:19p translocation*	XX XX XX XX *0% excess*	XX XX XX XX *0% excess*	XX XX XX XX *100% excess*

Legend: X = inactive X; X = active X; % excess repressor; NA = not applicable

Shown is the amount of XIST repressor received by the X chromosome, depending on the number of copies of 19p involved in the interaction in three individuals: 46 XX *(female with two normal X chromosomes)*; 46, XX; 19p dup interstitial (*female abortus with two copies of 19p, but one has a tandem duplication)*; and 46 XX(−3, +der3 t(3:19) *(female born with 3 separate copies of chromosome 19p, – two normal plus one on the 3:19 translocation chromosome).*

During X inactivation, one, and only one, X chromosome needs to be protected by chromosome 19. There are several possible ways to accomplish this (see Table 2): It may be that only one of the copies of critical genes on 19p is functional and the amount of repressor generated by one allele is sufficient to repress a single *XIST* locus. However, if the product from only one 19p was sufficient then in the case of the interstitial duplication, only half the cells would receive too much *XIST* repressor. This may not be enough to interfere with embryonic development. Further, if only one chromosome 19 were needed, the 19p trisomy due to unbalanced translocation would have no adverse effect on *XIST* repression (Table 2).

Alternatively, if gene product from *both* chromosomes 19p were needed to repress one *XIST* locus, then the female with a duplication affecting one chromosome 19p, would always provide too much repressor, hence inactivating both *XIST* loci, which would be non-viable, as observed (Table 2). If all chromosomes 19p were fully expressed, then having three chromosomes 19p would always produce too much repressor, and would be non-viable, which is not observed.

The new DECIPHER data do not distinguish between one or two functional 19p chromosomes. However, they tell us that if all 19p chromosomes in the cell produce the repressor, the level of product is not excessive in every cell. The fact that females (cases *394,820* and *395,096* in Table 2) with the relevant regions of chromosomes 19 present on three separate chromosomes were liveborn, suggest that each copy fires stochastically, thus reducing the chance that the repressor comes from all three 19ps in sufficient amounts to repress *both XIST* genes. That these females survive, means that either they have only one active X, or they are mosaic for one and two active X’s – which would not happen if all three 19p chromosomes were expressed in every cell.

My interpretation that the relevant gene products on chromosome 19 are generated asynchronously reflects the recent realization that expression of many genes occurs stochastically [23]. It is not yet known if one or both 19 alleles are transcribed in early embryos, yet, many genes on human autosome are reported to be mono-allelically expressed, especially in single cell assays [23,24]. Although these data often reflect the asynchrony of transcriptional bursting at many autosomal loci [25], some genes are known to be transcribed from only one of the two alleles. In some cases, one parental allele is imprinted by DNA methylation and consequently it is not expressed [26]. In the case of the immunoglobulin and olfactory genes, abundant evidence indicates that only one allele is expressed [27,28]; yet despite many studies, the details of allelic exclusion are not yet well understood. In the stochastic model, the amount of *XIST* repressor delivered in the *normal* female varies from cell to cell. contributing to the loss of females prior to implantation. If the product of both alleles is required then most cells receive the right amount, but a few have too little or too much. And some females might die in the process, contributing to the loss of that sex, prior to implantation [6].

My suggestion of a physical interaction between 19 and X is also in line with current views on higher order genome organization. Most intergenic interactions are thought to occur within genes on the same chromosome by means of topologically associating domains, TADs [29]. There is increasing evidence that interactions between two different chromosomes occur in hubs within the nucleus – reminiscent of the nucleoli that consist of a hub of acrosomic chromosomes, which, together, orchestrate the transcription of ribosomal genes [28]. Because the transcription of X-linked genes is up-regulated to equal that of autosomes [30], clearly X-autosomal interactions do occur.

Studies of chromosome positions in interphase nuclei suggest that they correlate with transcriptional activity [31,32] and that homologues may occupy different positions, depending on their transcriptional activity [31]. It is tempting to speculate that the future active X physically interacts with a pair of chromosomes 19p during early development. The reported studies of chromosome 19 compare its position to that of the less expressed chromosomes 18 [33,34] These data suggest that in several cell types the position of both 19s are in the interior of the nucleus, whereas the two relatively less active 18s are closer to the nuclear lamina [33]. Yet, the position of chromosomes X and 19 during the relevant stage of embryogenesis (prior to when X inactivation occurs), and its relation to transcription or epigenetic changes has not been shown. Looping out from chromosomes has been implicated in inter-chromosomal interactions [32] but looping studies have not been carried out during embryonic staging.

Three discrete copies of 19p are also reminiscent of other human trisomies. In individuals with Down syndrome, who are trisomic for chromosome 21, the expression of genes on that triplicated chromosome depend on their burst frequencies; only the highly expressed genes are overexpressed relative to diploid cells in single cell assays; however, in bulk assays all three genes may be expressed [35]. Yet, HiC studies for chromatin interactions in trisomy 21 show that some chromatin changes compartments from B to A, or A to B, affecting gene expression (Stylianos Antonarakis, personal communication). Conceivably, 19p trisomies interact with chromatin differently than interstitial 19p duplications.

The cytological observations from DECIPHER suggest but cannot tell us that both alleles of chromosome 19p genes are normally expressed in its interaction with the *XIST* locus. However, the ability of females with three discrete copies of 19p to survive implantation does make it is highly unlikely that all three 19p alleles are transcribed in every cell (Table 1). Also, the unique vulnerability of females with interstitial duplications of 19p suggests that at the time of the interaction between chromosome 19p and the human X, both X chromosome homologs must be close enough to one another to receive the repressor overflow.

Studies of chromosomal interactions in nuclear space, during preimplantation development, may begin to answer these questions. When the critical repressor on 19p is identified, one can demonstrate its interaction with *XIST* by studies of relevant cells at the right moment of human development. Until then, one could start by examining the number of chromosomes 19 in proximity with one X, as well as their position in the nucleus prior to upregulation of the non-repressed *XIST* locus, and the onset of X inactivation in preimplantation human embryos.

## Web resources

The URLs for data presented herein are as follows:

DECIPHER, https://decipher.sanger.ac.uk/
